# Highly Sensitive Multi-Channel Biosensor for Low-Interference Simultaneous Detection

**DOI:** 10.3390/nano13020246

**Published:** 2023-01-06

**Authors:** Jiapeng Su, Gongli Xiao, Hongyan Yang, Jiayu Chen, Haiou Li, Xingpeng Liu, Yunhan Luo, Jianqing Li

**Affiliations:** 1 Guangxi Key Laboratory of Precision Navigation Technology and Application, Guilin University of Electronic Technology, Guilin 541004, China; 2 Guangxi Key Laboratory of Optoelectronic Information Processing, School of Optoelectronic Engineering, Guilin University of Electronic Technology, Guilin 541004, China; 3 College of Science & Engineering, Jinan University, Guangzhou 510632, China; 4 Guangdong-Hong Kong-Macao Joint Laboratory for Intelligent Micro-Nano Optoelectronic Technology, Macau University of Science and Technology, Macau 999078, China

**Keywords:** multi-channel simultaneous detection, surface plasmon resonance, fiber optic sensor

## Abstract

In this paper, we propose a multi-channel photonic crystal fiber sensor, which adopts dual-polarization and multiple materials to effectively reduce the mutual interference between channels and enhance the surface plasmon resonance, thus achieving simultaneous detection of a multi-channel with low interference. Four channels are polished around the cylindrical fiber, and then different metal films (gold or silver) and plasmonic materials (titanium dioxide, thallium pentoxide, or graphene) are added to the sensing area of each channel. All channels detect refractive indices in the range of 1.34 to 1.42. The sensing performance of the fiber optic sensor was numerically investigated using the full vector finite element method. After the optimization of structural parameters, the maximum wavelength sensitivity of channel-1, channel-2, channel-3, and channel-4 are 49,800 nm/RIU, 49,000 nm/RIU, 35,900 nm/RIU, and 36,800 nm/RIU, respectively. We have theoretically analyzed the sensor’s capabilities for partial bio-detection and simulated its detection capability with a wavelength sensitivity of 11,500 nm/RIU for normal red blood cells and 12,200 nm/RIU for MCF-7 cancerous cells. Our proposed sensor has a novel design, can detect multiple channels simultaneously, has strong anti-interference capability and high sensitivity, and has good sensing characteristics.

## 1. Introduction

Surface plasmon resonance (SPR) is an electromagnetic phenomenon resulting from the oscillation of free electrons caused by the total reflection of light at the interface between metal and a dielectric material [1]. Photonic crystal fiber (PCF) is usually made of fused silica, polymers, or plastics as the background material and has periodic or nonperiodic arrays of microtia extending along the full length of the fiber [2]. In recent years, the SPR–PCF sensor proposed by applying SPR technology to PCF had numerous applications in the sensing field due to its small size, high sensitivity, and reusability [3].

Multi-channel sensors not only enable the simultaneous detection of multiple analytes and greatly improve the detection efficiency of the sensors, but also effectively reduce the detection error caused by time or operation of biological information [4]. In the SPR–PCF sensors, multi-channel sensors can be divided into two categories. One is the use of different materials to make the detection range of each channel different [5]. In 2020, Pibin Bing et al. proposed a dual-channel simultaneous detection of photonic crystals in an analytical fiber optic sensor with two channels for gold (Au) and silver (Ag) sensing metals, and two channels with detection wavelength sensitivities (WS) of 11,600 nm/RIU and 10,600 nm/RIU [6]. In 2021, K. M. Mustafizur Rahman et al. proposed a simple and highly sensitive multi-channel hexagonal photonic crystal fiber sensor based on the SPR refractive index (RI), which uses different materials (Au, TiO_2_, and Ta_2_O_5_) combined with each other to achieve a maximum WS of 38,100 nm/RIU, 36,100 nm/RIU, and 45,800 nm/RIU for three-channel sensing, respectively [7]. However, there are obvious shortcomings in this type of sensing. When the RI of multi-channel analytes are similar, it is not sufficient to distinguish each channel, and the interference between channels is too great to complete simultaneous detection. Another is the dual-polarization sensor, which uses the dual-polarization of light to accomplish multi-channel detection [8]. In 2018, Md. Rabiul Hasan et al. proposed an SPR biosensor based on a dual-polarized spiral photonic crystal fiber. The sensor achieves a maximum WS of 3000 nm/RIU and 4000 nm/RIU detection using different polarizations [9]. In 2021, Iddrisu Danlard et al. proposed a dual-polarized quasi-D-type plasma PCF, which detected RI and temperature with dual-polarization and a maximum WS of 5000 nm/RIU and 3 nm/°C for both channels [10]. Similarly, dual-polarization multichannel sensors have the disadvantage that the number of channels is severely limited due to the limitation of polarized light [11]. Therefore, to avoid the defects of the above two types of sensors, we combine a multi-material with dual-polarization to compensate for the singularity of dual polarization detection and the limitation of multi-material detection of similar RI.

In this work, we designed and investigated a four-channel SPR–PCF sensor based on dual-polarized and different materials. The sensor is constructed by polishing four symmetrical planes around the cylindrical fiber and coating different metals (Au and Ag) and different plasmonic materials (TiO_2_, Ta_2_O_5_, and graphene) on different sides, thus building four detection channels. By studying materials, structures, and light sources, the interference between the channels of the sensor is greatly reduced while ensuring high sensitivity. After the optimization of the structural parameters, the sensor model with superior performance was obtained. In addition, we have theoretically analyzed the partial bio-information (blood, cancer, virus) detection capability of the sensor, providing a new research idea for the simultaneous detection of bio-information.

## 2. Sensor Design and Theory

**Figure 1:**
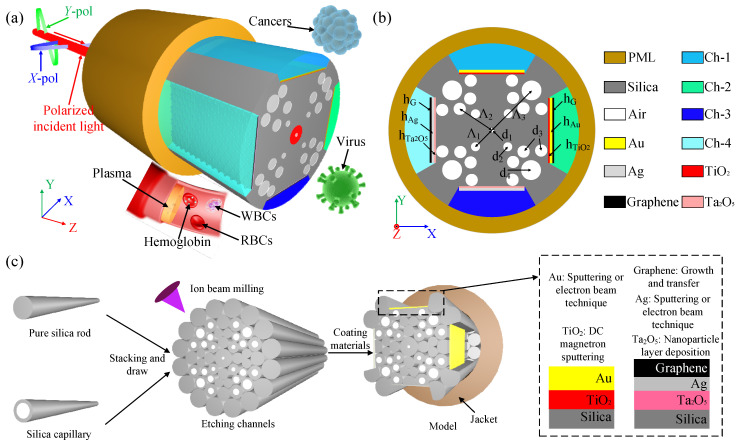
Sensor Model. (**a**) Three-dimensional model; (**b**) Two-dimensional cross-sectional view of the sensor; (**c**) Manufacturing model.

Figure 1a,b shows the sensor model. The air-holes in the sensor are arranged in a circle in four layers. In the center of the fiber is an innermost air-hole, whose diameter is d_1_ = 0.4 μm. The distance between the second inner layer, the second outer layer, and the outermost air-holes and the innermost air-holes are Λ_1_ = 2 μm, Λ_2_ = 4 μm, and Λ_3_ = 6 μm respectively. All three layers of air-holes are distributed in the four corners of the fiber, the second inner layer has four air-holes of diameter d_2_ = 0.6 μm; the second outer layer has eight air-holes of diameter d_3_ = 1.2 μm; the outermost layer has four air-holes of diameter d_4_ = 1.6 μm, both sides of which have a diameter of d_3_ = 1.2 μm air-holes. Polishing the top, bottom left and right of the fiber is performed to form channel-1 (Ch-1), channel-2 (Ch-2), channel-3 (Ch-3), and channel-4 (Ch-4). In the casting plane of Ch-1 and Ch-2, a layer of TiO_2_ with thickness h_TiO_2__ = 10 nm is first applied, and then covered with a layer of Au with thickness h_Au_ = 50 nm; In the casting plane of Ch-3 and Ch-4, a layer of Ta_2_O_5_ with thickness h_Ta_2_O_5__ = 10 nm is first applied, and then covered with a layer of Ag with thickness h_Ag_ = 50 nm; Because the fiber structure is symmetric, the performance of Ch-1 at *Y*-polarization (*Y*-pol) and Ch-2 at *X*-polarization (*X*-pol) are the same. Therefore, to use each channel more fully, another graphene layer of thickness h_G_ = 0.34 nm is added on the outside of the Au layer of Ch-2, and similarly, a graphene layer is added to Ch-4.

To better implement and validate the described optical fiber sensor, we analyzed and studied the fabrication of the fiber optic sensor. Although the structure we propose is complex, the process can be upgraded to make it more accurate. As shown in Figure 1c, pure silica rods and silica capillaries of different sizes are stacked regularly and stretched at high temperature. Then, we will construct four sensing channels, and the focused ion beam milling and femtosecond laser micromachining are used because the conventional polishing process is difficult to polish four planes on one fiber [12]. Finally, the techniques in Figure 1c are used to coat the sensing area with different talents. In addition, a layer of jacket was wrapped at the outermost periphery [13].

A variety of materials are involved in the paper, and we use the RI or dielectric constant of the material to express the properties of each material. The RI of silicon dioxide can be expressed by the Sellmeier formula [14], the dielectric constants of Au and Ag can be expressed by the Drude–Lorenz model [15,16] and the RI of TiO_2_ can be expressed as: [17]

(1) $$n_{{\mathrm{TiO}}_{2}}=5.913+\frac{2.441\times 10^{7}}{\left(\lambda^{2}-0.803\times 10^{7}\right)}$$

where $n_{{\mathrm{TiO}}_{2}}$ is the RI of TiO_2_, λ is the wavelength of the incident light. The RI of Ta_2_O_5_ can be expressed as: [18]

(2) $$n_{{\mathrm{Ta}}_{2}O_{5}}=1.88+\frac{178.4\times 10^{2}}{\lambda^{2}}+\frac{527\times 10^{7}}{\lambda^{4}}$$

where $n_{{\mathrm{Ta}}_{2}O_{5}}$ is the RI of Ta_2_O_5_. The RI of graphene can be expressed as: [19]

(3) $$n_{G}=3.0+i\frac{C}{3}\lambda$$

where $n_{G}$ is the RI of graphene and C ≈ 5.446 μm^−1^. The RI of air is normally 1. The constraint loss is a very important part in the analysis of sensing performance, and its equation is expressed as: [20]

(4) αLoss(λ)=40πλln10Im(neff)×104

where Im(neff) is the imaginary part of the effective RI.

The level of sensitivity is an important indicator of sensor performance. WS and amplitude sensitivity (AS) is usually used to reflect the performance of the sensor. The expression of WS is: [21]

(5) $$S_{\lambda }=\frac{\Delta \lambda_{peak}}{\Delta N}$$

where $\Delta \lambda_{peak}$ is the offset of the resonance wavelength (RW) between adjacent RI, ΔN is the amount of change in RI of the analyte. AS is strongly related to the propagation loss, which is expressed as: [22]

(6) $$S_{A}=-\frac{1}{\alpha \left(\lambda ,N\right)}\frac{\delta \alpha \left(\lambda ,N\right)}{\delta N}$$

where α(λ,N) represents the loss value at the incident light wavelength λ when the RI is N. Variable δN represents the difference in RI between adjacent analytes. Variable δα(λ,N) represents the difference of loss values at adjacent RIs under the same incident light wavelength λ. The figure of merit (FOM) is also one of the important indicators of sensor performance, and its expression is: [23]

(7) $$FOM=\frac{S_{\lambda }}{FWHM}$$

where $S_{\lambda }$ is WS, FWHM is the full width at half maximum, which describes the wave characteristics.

## 3. Simulation Results and Discussions

To ensure the sensor design is reasonable and the calculation is accurate, we analyze the model based on the finite element method (FEM) and use COMSOL Multiphysics software simulation and calculate.

### 3.1. Modes Analysis

**Figure 2:**
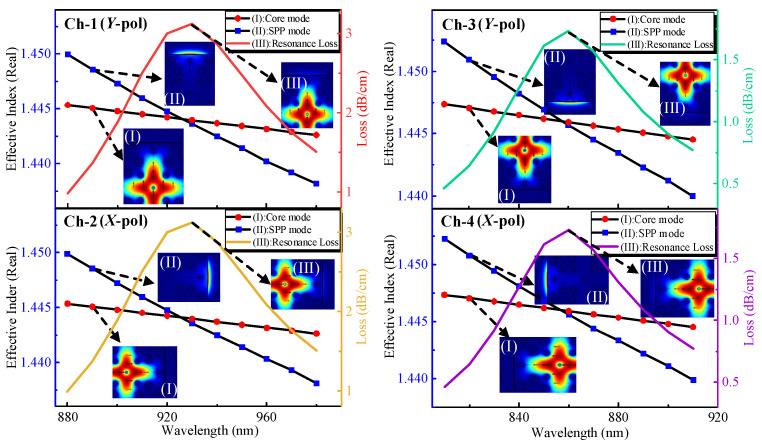
Modes analysis and dispersion relationship of each channel in RI 1.40.

Light propagation in optical fibers can be solved by the fluctuation equation, and the longitudinal and transverse field solutions in optical fibers are different, so this requires a mode analysis of the fiber. Figure 2 shows the modes analysis and dispersion relationship of each channel. The RI of the analyte is 1.40 for all four channels, and all parameters are the same as the model in Figure 1, except that the graphene layer is not added to Ch-2 and Ch-4. When most of the light propagates in the center of the fiber, the polished detection plane has no light intensity, and this mode is called the core mode. Conversely, when there is almost no light in the core and most of the energy is gathered in the detection plane, this mode is called SPP mode. The real part of the effective refraction of the two modes varies with the wavelength of the incident light [24]. When the real parts of these two modes are equal, a resonant mode occurs, which is represented by a loss curve. In Figure 2, I, II, and III show the core mode, SPP mode, and resonant mode, respectively. Currently, the fiber core has more energy and the detection surface also has energy. Different detectors have different refractive indices, and the wavelengths of the resonance occurrences are different, and the detection of analytes is achieved by analyzing different RW.

It can also be found from Figure 2 that the Ch-1 with *Y*-pol and Ch-2 with *X*-pol of loss curves are the same. This is because the fiber optic sensor is symmetrical, Ch-1 and Ch-2 are only different in orientation. Ch-3 and Ch-4 have the same principles. For better differentiation and to detect the biological information of large molecules, we added graphene layers to the metal surfaces of Ch-2 and Ch-4.

### 3.2. Interference Analysis

**Figure 3:**
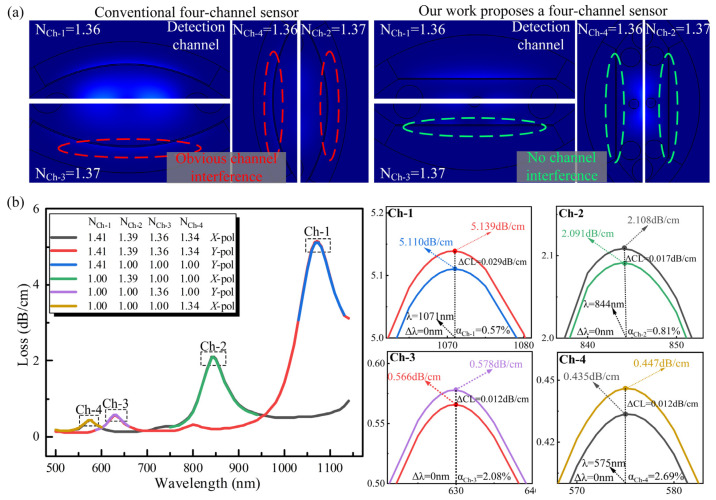
Inter-channel interference analysis. (**a**) Comparison of multi-channel sensing with different structures. (**b**) Multi-channel simultaneous detection and multi-channel separate detection.

The way to reduce the mutual interference between multiple channels is a major problem of multi-channel detection [25]. We effectively reduce the influence of each channel from three aspects: light source, structure, and material. The combination of different polarized light and detection planes can effectively reduce the interference between channels; the RW is changed by different materials under the same polarized light, thus further reducing the interference. Figure 3a shows the comparison between our proposed multichannel sensor model and a conventional fiber optic sensor. Although the conventional sensor is simple in process, there is obvious interference between the channels due to the limitation of the structure, which will affect the accuracy of detection. Our proposed sensor greatly reduces this type of interference by optimizing the structure and materials.

Figure 3b shows the multi-channel detection analysis. First, we added analytes with refractive indices of N_Ch-1_ = 1.41, N_Ch-2_ = 1.39, N_Ch-3_ = 1.36, and N_Ch-4_ = 1.34 in Ch-1, Ch-2, Ch-3, and Ch-4 simultaneously and detected them with *X*-pol light and *Y*-pol light, respectively, and obtained the corresponding loss spectra. Then we added the corresponding analytes to one channel only, and the other channels did not have any added analytes. The above method was used to compare and analyze the interference when multiple channels were detected simultaneously. The ΔCL indicates the amount of change in resonance loss in both cases. The α is the percentage of ΔCL over the loss of one channel, which indicates the interference of simultaneous detection of multiple channels to a single channel. From Figure 3b, it can be obtained that the Δλ of all four channels is 0, which indicates that there is no change in the RW between simultaneous detection of multiple channels and independent detection of a single channel. From the loss point of view, the larger the RI of the analyte, the smaller the interference between multiple channels, when the RI of the analyte takes the minimum value of 1.34, the maximum interference between multiple channels only accounts for 2.69% of the single channel detection loss. The above data can prove that our proposed sensors have less mutual interference when detected simultaneously.

### 3.3. Materials Selection

**Figure 4:**
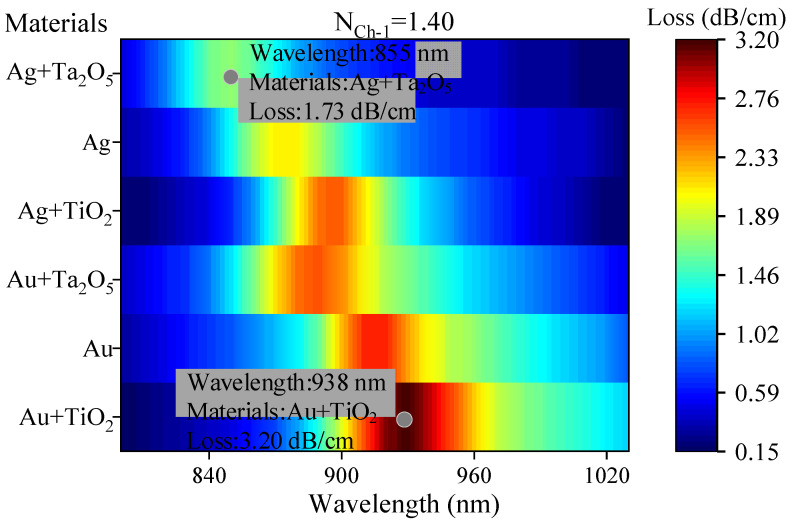
Selection and analysis of detection plane materials.

In SPR–PCF sensors, the choice of materials is crucial [26]. In this paper, we choose Au and Ag as SPR metals, and TiO_2_ and Ta_2_O_5_ as plasmonic materials, and analyze these materials in the proposed model. This is shown in Figure 4. The analytes in Ch-1 have a RI of 1.40. The loss curves of the detection planes of six different combinations were analyzed by combining Au, Ag, TiO_2_, and Ta_2_O_5_ with each other, both with a thickness of 50 nm for Au and Ag, and both with a thickness of 10 nm for TiO_2_ and Ta_2_O_5_. From Figure 4, it is seen that the RW and resonance loss are shifted the most in these two cases of Au + TiO_2_ and Ag + Ta_2_O_5_, which also means the least interference between the two. Therefore, the two types of Au + TiO_2_ and Ag + Ta_2_O_5_ will be used in the selection of each channel material.

### 3.4. Detection Area Optimization

**Figure 5:**
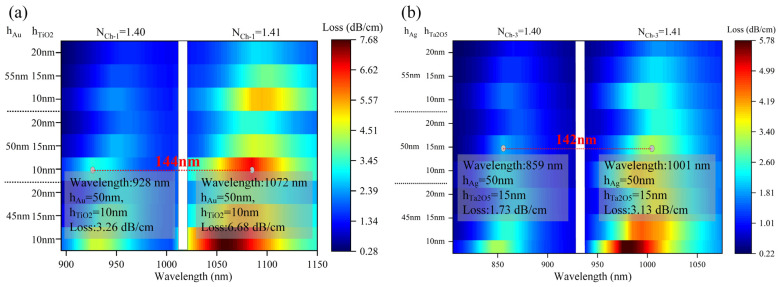
Effect of different thicknesses of materials on sensing performance. (**a**) Thickness analysis of Au layer and TiO_2_ layer in Ch-1; (**b**) Thickness analysis of Ag layer and Ta_2_O_5_ layer in Ch-3.

We have determined the materials used in the sensing area, but the thickness of each material also has a significant impact on the detection results. The thickness of each material is now compared and analyzed. Since the fiber is symmetrical, the data of Ch-1 and Ch-2 are the same, as well as Ch-3 and Ch-4, so here Ch-1 and Ch-3 are analyzed first. Detailed data analysis is shown in Figure 5. In Ch-1, when h_Au_ = 50 nm and h_TiO_2__ = 10 nm, the maximum sensitivity is 14,400 nm/RIU; In Ch-3, when h_Au_ = 50 nm and h_TiO_2__ = 15 nm, the maximum sensitivity is 14,200 nm/RIU. Therefore, we can determine that the thickness of the Au layer is 50 nm, the thickness of TiO_2_ is 10 nm, the thickness of Ag is 50 nm, and the thickness of Ta_2_O_5_ is 15 nm as the optimal structure by the above analysis.

**Figure 6:**
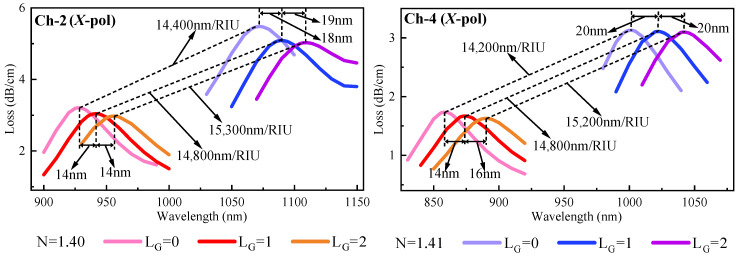
Variation of loss curves for introducing 0 to 2 layers of graphene on the metal surface of Ch-2 and Ch-4.

Ch-1 and Ch-2 have the same sensing area, and Ch-3 and Ch-4 have the same sensing area. To better differentiate the channels and to better detect large molecules during bioassay, we will coat the metal surfaces of Ch-2 and Ch-4 with graphene. The number of graphene layers will also have an impact on the detection performance. Figure 6 shows the variation of 0 to 2 layers of graphene on Ch-2 and Ch-4. Although the sensitivity improvement brought by 2-layer graphene is the largest, the RW offset it produces is also the largest, and too large an offset will cause greater interference to other channels when detecting. Therefore, we chose to add one layer of graphene on the metal surface of Ch-2 and Ch-4 with a single layer thickness of 0.34 nm.

### 3.5. Sensor Performance

**Figure 7:**
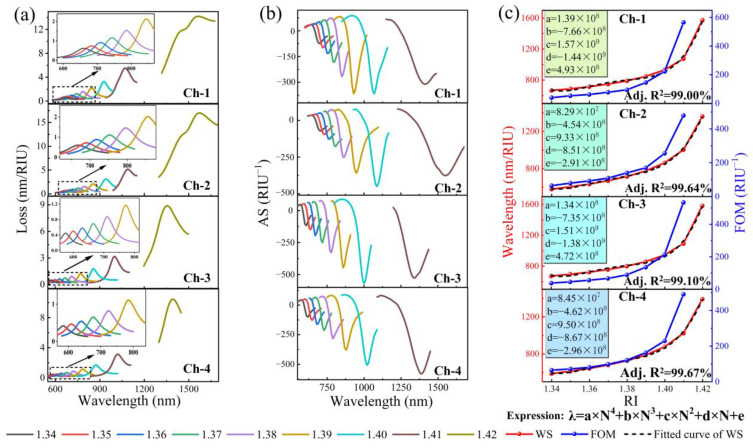
Comprehensive performance analysis of the proposed multichannel sensor. (**a**) Loss curve of each channel; (**b**) AS of each channel; (**c**) FOM and RW fitting curves for each channel.

As a result of the above analysis, we have determined the scientific and optimal model of the proposed SPR–PCF sensor. Now, we are going to simulate and analyze the overall performance of the optimal model. The proposed and reasonably optimized sensor parameters are selected, and the detection range of the analytes for the four channels is 1.34 to 1.42. In Figure 7, we have derived the loss curves, WS, AS, FOM, and RW fitting curves for the four channels by simulation calculation and analysis. The detailed data are shown in Table 1. In addition, we also curve-fitted the resonant wavelengths of each channel, and the adjusted R^2^ of each curve was greater than 99.00%. This gives us a better understanding of the sensor performance.

To illustrate the proposed sensor more strongly, we compared it with sensors recently proposed by other researchers. Detailed comparison data are shown in Table 2. Although models 1 to 3 achieve multi-channel detection, the detection sensitivity is not high. Model 4 has a high sensitivity, but it cannot detect analytes with similar RI at the same time because of the interference between channels, as described by its authors in the original paper. Compared with the above models, our proposed model ensures high detection performance and designs a multi-channel simultaneous detection sensor with low interferences from the material, polarization, and structure perspectives, which is an excellent design at this stage.

### 3.6. Bio-Detection Performance

The above analysis has sufficiently proved that the sensor we designed is good enough, and now the analytes are replaced with biological information for the actual. Three groups of bioinformatics are examined; the first is blood information, which contains red blood cells (RBCs), white blood cells (WBCs), plasma, and hemoglobin [30]. The second group is the effect of the malaria virus on erythrocytes, including RBCs normal, ring, trophozoite, and Schizont [31]. The third group is normal and cancerous cells, which includes HeLa normal and cancerous and MCF-7 normal and cancerous [7]. Detailed test performance is shown in Table 3. To express the detection of biological information more accurately, we used the average WS, average AS, and average FOM to describe the detection performance. The sensor can detect multiple biological information at the same time, for example, to separate the blood into RBCs, WBCs, plasma, and hemoglobin by the centrifuge. Put the four detection substances into the corresponding channel, you can detect the four detection substances at the same time, to avoid the influence of time or other interference on the biological information, to ensure the accuracy of the detection. The sensor’s ability to detect biological information is superior to that of similar sensors. In addition, to ensure that the performance of our proposed sensor is excellent, the results of the biological information detected by the sensor are compared with previous work, and Table 4 shows the detailed data. From the table, it can be seen that our proposed sensor is superior to its counterparts in both blood component detection and virus detection.

## 4. Conclusions

In summary, a biosensor with simultaneous multi-channel detection is proposed. The interference between channels is reduced by using different materials (Au, Ag, TiO_2_, Ta_2_O_5_, and graphene) and polarization (*X*- and *Y*-pol) to achieve highly sensitive multi-channel biosensors for low-interference simultaneous detection. The detected RI range is from 1.34 to 1.42, and the maximum WS of the four channels are 49,800 nm/RIU (Ch-1), 49,000 nm/RIU (Ch-2), 35,900 nm/RIU (Ch-3), and 36,800 nm/RIU (Ch-4). In addition, the interference between the sensor channels is less than 2.69%, which has good anti-interference ability. The detection capability of this sensor is superior to that of the same type of sensors at this stage. Additionally, we conducted a theoretical analysis of the sensor for the detection of partial blood information, cancer, and viruses, which may simultaneously detect biological information and enhance detection accuracy due to decreased channel interference. The bio-detection capability is also superior to the same type of sensors. The sensor has high sensitivity, simultaneous multi-channel detection, low interference, flexible application, and high applicability, and has good research value and application prospects in sensor detection and bioinformatics detection.

## Figures and Tables

**Table 1:** Performance analysis of the proposed multi-channel sensor.

	RI	RW (nm)	Loss (dB/cm)	WS (nm/RIU)	AS (RIU^−1^)	FOM (RIU^−1^)		RI	RW (nm)	Loss (dB/cm)	WS (nm/RIU)	AS (RIU^−1^)	FOM (RIU^−1^)
	1.34	661	0.615	2200	−75	36		1.34	575	0.446	2500	−118	62
	1.35	683	0.739	2700	−100	44		1.35	600	0.500	3000	−150	77
	1.36	710	0.911	3400	−132	54		1.36	630	0.578	3600	−198	90
	1.37	744	1.159	4200	−183	65		1.37	666	0.695	4600	−251	107
Ch-1	1.38	786	1.530	5800	−268	87	Ch-2	1.38	712	0.874	6100	−330	138
	1.39	844	2.132	8400	−367	134		1.39	773	1.169	8600	−448	168
	1.40	928	3.200	14,400	−368	212		1.40	859	1.732	14,200	−572	254
	1.41	1072	5.580	49,800	−312	534		1.41	1001	3.131	35,900	−530	479
	1.42	1570	14.193	NA	NA	NA		1.42	1360	8.890	NA	NA	NA
	1.34	668	0.597	2300	−73	36		1.34	575	0.415	2600	−101	63
	1.35	691	0.715	2700	−96	44		1.35	610	0.465	3000	−128	70
	1.36	718	0.877	3400	−128	54		1.36	640	0.540	3700	−161	79
	1.37	752	1.098	4400	−180	65		1.37	677	0.650	4700	−216	96
Ch-3	1.38	796	1.456	5900	−253	87	Ch-4	1.38	724	0.821	6100	−284	120
	1.39	855	2.021	8700	−362	134		1.39	785	1.100	8900	−382	162
	1.40	942	3.036	14,800	−453	212		1.40	874	1.665	14,800	−510	228
	1.41	1090	5.087	49,000	−378	534		1.41	1022	3.110	36,800	−584	491
	1.42	1580	16.670	NA	NA	NA		1.42	1890	10.44	NA	NA	NA

**Table 2:** Performance comparison of the proposed multi-channel sensor with existing sensors.

Design	Wav. Range (nm)	RI Range	Max WS (nm/RIU)	Max AS (RIU^−1^)	Max FOM (RIU^−1^)
Dual Channels Au/Ag [27]	400–800	1.33–1.37	2500 (Ch-1)	NA	NA
			3083 (Ch-2)		
Dual Channels Au [28]	813–1100	1.30–1.40	1000 (Ch-1)	0.5 (Ch-1)	NA
			3750 (Ch-2)	40 (Ch-2)	
Three Channels Au [29]	500–1045	1.33–1.35	2000 (Ch-1)	95 (Ch-1)	34 (Ch-1)
		1.36–1.38	3000 (Ch-2)	184 (Ch-2)	65 (Ch-2)
		1.39–1.42	18,000 (Ch-3)	427 (Ch-3)	200 (Ch-3)
Three Channels Au and Ta_2_O_5_/TiO_2_ [7]	690–1440	1.37–1.42	38,100 (Ch-1)	582 (Ch-1)	635 (Ch-1)
	690–1250	1.37–1.42	21,600 (Ch-2)	969 (Ch-2)	432 (Ch-2)
	690–1650	1.36–1.42	45,800 (Ch-3)	1230 (Ch-3)	572 (Ch-3)
Four Channels Au/Ag and TiO_2_/Ta_2_O_5_/graphene (Our work)	661–1570	1.36–1.42 (Ch-1)	49,800 (Ch-1)	312 (Ch-1)	565 (Ch-1)
	668–1580	1.36–1.42 (Ch-2)	49,000 (Ch-2)	378 (Ch-2)	534 (Ch-2)
	575–1360	1.36–1.42 (Ch-3)	35,900 (Ch-3)	530 (Ch-3)	479 (Ch-3)
	584–1390	1.36–1.42 (Ch-4)	36,800 (Ch-4)	584 (Ch-4)	491 (Ch-4)

**Table 3:** Performance of the proposed multi-channel sensor in bioinformatics detection.

Type of Test	RI	RW (nm)	Loss (dB/cm)	WS (nm/RIU)	AS (RIU^−1^)	FOM (RIU^−1^)
Plasma (Ch-3)	1.35	600	0.500	3000	−132	70
Hemoglobin (Ch-1)	1.38	786	1.530	5500	−221	84
WBCs (Ch-4)	1.36	640	0.540	3500	−143	75
RBCs (Ch-2)	1.40	942	3.036	11,500	−398	176
RBCS at ring phase (Ch-1)	1.395	877	2.608	8400	−371	147
RBCS at trophozoite phase (Ch-3)	1.383	726	0.958	5900	−310	130
RBCS at Schizont phase (Ch-4)	1.373	689	0.700	4600	−197	102
HeLa Normal (Ch-4)	1.368	666	0.619	4000	−176	90
HeLa Cancerous (Ch-1)	1.392	855	2.246	8100	−346	156
MCF-7 Normal (Ch-3)	1.387	747	1.037	6300	−350	144
MCF-7 Cancerous (Ch-2)	1.401	953	3.165	12,200	−409	185

**Table 4:** Bioinformatic detection comparison of the proposed multi-channel sensor with the existed sensors.

Type of Test	RI	Reference	RW (nm)	Δλ_peak_ (nm)	Reference	RW (nm)	Δλ_peak_ (nm)
Plasma	1.35	[31]	666	30	Our work	600	40
WBCs	1.36		696	88		640	146
Hemoglobin	1.38		784	248		786	156
RBCs	1.40		1032	NA		942	NA
RBCS at Schizont phase	1.373	[32]	698	48	Our work	689	37
RBCS at trophozoite phase	1.383		746	90		726	151
RBCS at ring phase	1.395		836	100		877	65
RBCS at normal	1.40		936	NA		942	NA
HeLa Normal	1.368	[7]	750	120	Our work	666	189
HeLa Cancerous	1.392		870	NA		855	NA
MCF-7 Normal	1.387		750	80		747	206
MCF-7 Cancerous	1.401		830	NA		953	NA

## Data Availability

Data sharing not applicable.
